# Development, validation, and pilot implementation of the minimum datasheet for a domestic violence registry system: The case of a developing country

**DOI:** 10.1371/journal.pone.0261460

**Published:** 2021-12-31

**Authors:** Shabnam Iezadi, Kamal Gholipour, Ahmad Khanijahani, Mahasti Alizadeh, Bahram Samadirad, Hanie Azizi, Farzad Azizinia

**Affiliations:** 1 Hospital Management Research Center, Health Management Research Institute, Iran University of Medical Science, Tehran, Iran; 2 Social Determinants of Health Research Center, Department of Health Policy and Management, School of Management and Medical Informatics, Tabriz University of Medical Science, Tabriz, Iran; 3 Department of Health Administration and Public Health, John G. Rangos School of Health Sciences, Duquesne University, Pittsburgh, PA, United States of America; 4 Social Determinants of Health Research Center, Tabriz University of Medical Science, Tabriz, Iran; 5 Forensic Medicine research center, Iranian Legal Medicine Organization, Tehran, Iran; 6 Medical Education Research Center, Health Management and Safety Promotion Research Institute, Tabriz University of Medical Sciences, Tabriz, Iran; 7 School of medicine, Tabriz University of Medical Sciences, Tabriz, Iran; Victoria University of Wellington, NEW ZEALAND

## Abstract

**Background:**

Domestic violence (DV) is a universal issue and an important public health priority. Establishing a DV Registry System (DVRS) can help to systematically integrate data from several sources and provide valid and reliable information on the scope and severity of harms. The main objective of this study was to develop, validate, and pilot-test a minimum datasheet for a DVRS to register DV victims in medical facilities.

**Materials and methods:**

This study was conducted in two main phases. Phase one includes developing the datasheet for registration of DV in the DVRS. In phase two, the datasheet designed in the previous step was used in a pilot implementation of the DVRS for 12 months to find practical challenges. The preliminary datasheet was first developed using information on similar registry programs and guidelines of the World Health Organization (WHO) and then reviewed by four expert panels. Through a two-round Delphi technique, experts evaluated the instrument using the Content Validity Index (CVI) and Content Validity Ratio (CVR). The consistency of the responses was evaluated by test-retest analysis. Finally, two physicians in two forensic medical clinics registered the victims of physical and/or sexual violence perpetrated by a family member.

**Results:**

Preliminary datasheet consisted of 31 items. In the first round of Delphi, fifteen items had good content validity (I-CVI and CVR) and were kept, and seven items were moved to the next round. Also, in the first round of Delphi, experts suggested adding three items, including history of the violence, custody of the child, and custody of the elderly. All items evaluated in the second round were kept due to good CVR and CVI scores. As a result of Test-retest correlation coefficients for self-reprted items, two items including perpetrator’s alcohol and drug use status were excluded (r(30) = +.43, and +.38, p< .01, two-tailed, respectively). Finally, 24 items were included in the datasheet including 15 items for individuals’ characteristics (victims’ characteristics and perpetrators’ characteristics), eight items for incidents’ characteristics, and one item for past history of violence experience. A total of 369 cases were registered from September 23, 2019, to July 21, 2020. The majority of the reported cases were female (82%) and were 19–40 years old. No physical and/or sexual violence was reported from rural areas, which calls upon researchers to explore how services for detecting and treating the victims can be made accessible to these areas.

**Conclusion:**

DVRS can show trends in DV by age, sex, the context of the violence, and incidence characteristics at every point in time. This is particularly valuable in planning and prioritizing research areas and interventions for DV prevention. Additionally, DVRS can be linked to other disease registry programs which can contribute to continuity and coordination of care, and major research in the future. Although a DVRS can be a promising initiative in identifying the areas in need of urgent interventions, there is no guarantee for its proper implementation due to limited resources and other challenges.

## Introduction

Domestic violence (DV) is a global issue and an important public health priority causing more than one million deaths and even more injuries and disabilities among 15 to 44-year-old individuals annually [1, 2]. More importantly, DV impacts vulnerable populations more severely [3–6] and increases the risk of HIV, alcohol and drug abuse, abortion, low birth-weight, premature birth, suicide, and severe mental health problems [7]. According to the American Academy of Family Physicians (AAFP), DV is "the intentional intimidation or abuse of children, adults, or elders by a family member, intimate partner, or caregiver to gain power and control over the victim" [8, 9]. There has always been debate regarding this definition because of the limitation of "family" to traditional marriages. However, nowadays, it is widely accepted that a group of people related by birth, marriage, or adoption living together makes up a family [8, 9].

Abuse or violence can be in the forms of physical, sexual, emotional, or even financial. Statistics show that 35% of females worldwide have experienced non-partner sexual violence or intimate partner physical/sexual violence [7]. Approximately 38% of all female homicides are committed by their intimate partners. DV imposes a high burden on health systems and individuals, either directly or indirectly, and disproportionately impacts low- and middle-income countries [10]. About 10% of women experience sexual abuse by a partner in their life [11]. The adverse effects of domestic violence on children’s mental health, underutilization of needed healthcare services [12], cognitive functioning [13], and poor school performance [14] are well documented. Elderly abuse is associated with lower quality of life [15], higher risks of mortality and morbidity, and higher avoidable healthcare utilization rates [16].

There remain numerous challenges when studying DV and prevention strategies [17]. One main limitation of the studies on interpersonal violence, and particularly DV, is the use of self-reported data in estimates [18]. Therefore, it is difficult to determine if the participants have overestimated or underestimated the real intentionality and extent of the incidence and harms, particularly in countries like Iran with particular cultural pressures to keep family issues secret and within the family [19]. By emphasizing the systematic collection of data on violence incidents, public health introduces an evidence-based approach to deal with violence. This approach encourages collecting data at local, provincial, or national levels; research on causes and risk factors of violence; and development and implementation of preventive measures [2]. Therefore, establishing a DV Registry System (DVRS), which is a pinpoint of the public health approach, can help to systematically integrate data from several sources and provide valid and reliable information on the scope and severity of the harms.

There are some examples of violence registry systems from developed countries, including National Violent Death Reporting System (NVDRS) [20] established by the Centers for Disease Control and Prevention (CDC) and Injury Statistics Query and Reporting System (WISQARS™) [21] in the US, ACT Domestic and Family Violence Data Collection Project in Australia, [22] and so on. However, to our knowledge, there is no well-organized violence-related registry system in most developing countries and, to the scope of this study, from Iran. On the other hand, exemplified violence registry systems are government derived initiatives and we were not able to find a paper studying the method of developing and establishing these registry programs. However, violence is yet considered as a high-priority public health issue. Therefore, since the development and establishment of a DVRS, with respect to public health approach, is a relatively new and novel initiative, we reviewed other similar studies in the field of wound, trauma, or injury registry systems. These examples used different approaches to develop and/or validate the registry systems such as interphysician reliability assessment [23], registration of injured patients by a registrar for analysis [24], evaluation of data completeness [25], and exploring the problems of the registry system with regards to data entry, errors, and data collation [26]. Moreover, even these studies did not use a systematic approach to development and validation of the registry datasheets in an step-by-step process. There is also a great gap in knowledge on the development and validation of violence-related registry systems in Iran which urged us on conducting a comprehensive study to develop and validate a DVRS in Iran using a systematic approach.

East-Azerbaijan Province is the sixth most populated and industrial province in Iran. Although there are no robust and precise statistics on the prevalence of DV in Iran, organizational reports on the topic indicate that the prevalence and severity of the problem in this province are high and have shown increasing growth in the last decade [27]. After conducting robust qualitative research, experts from various disciplines such as forensic medicine, emergency medicine, community medicine, and health management planned to establish a DVRS as a strategy to collect and integrate data on DV incidents in order to facilitate research and planning on prevention of DV and its consequences [27]. The first step in establishing the DVRS is to develop a valid and reliable minimum datasheet. This study had two main objectives. First, to develop and validate a minimum datasheet to register victims of DV in medical facilities. Second, to pilot-test the designed DVRS in two Forensic Medicine Clinics (FMCs) to examine this datasheet’s practicality and real-life implementation.

## Method

**Fig 1:**
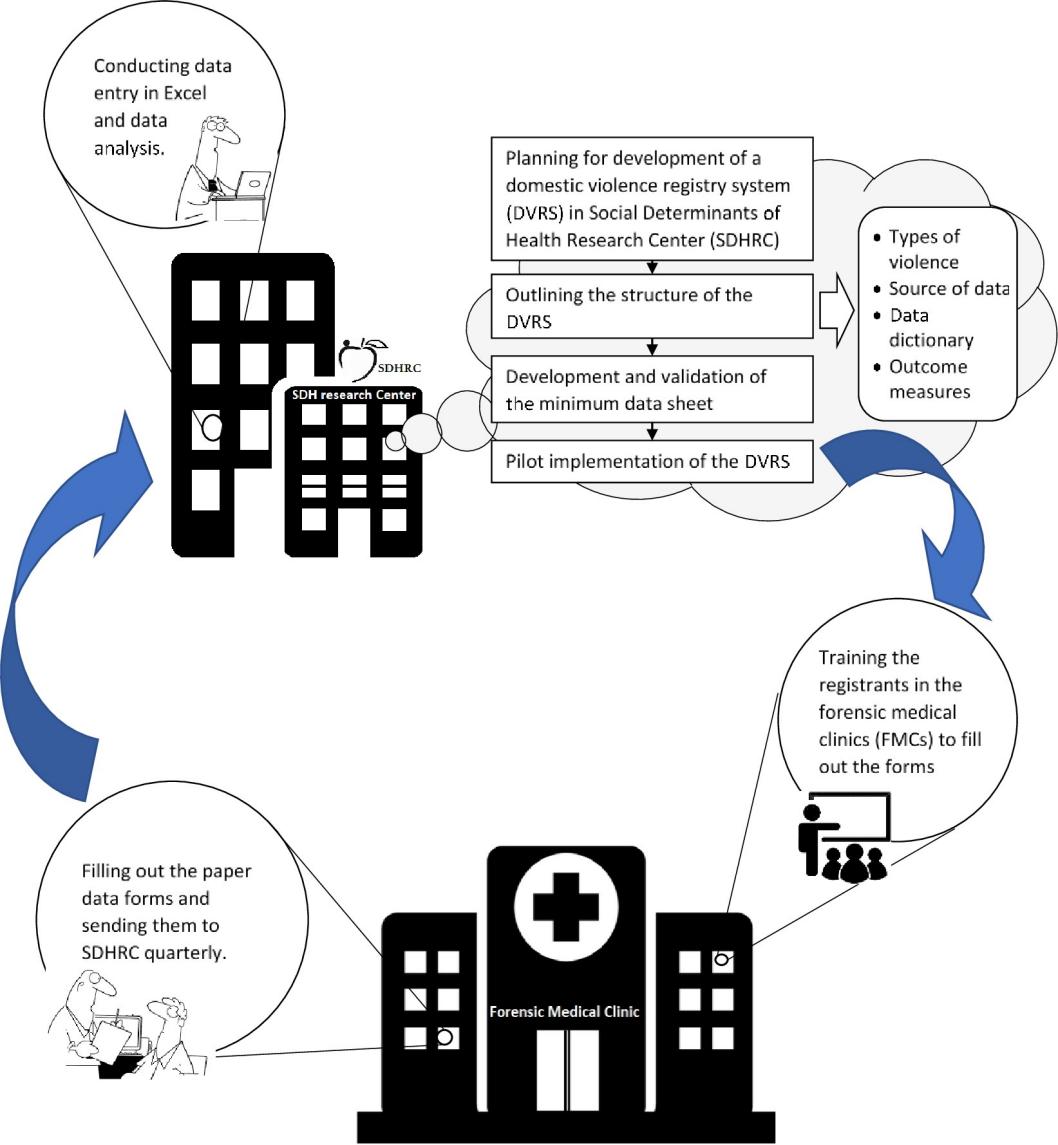
Process of the development and pilot implementation of the domestic violence registry system in East-Azerbaijan Province of Iran.

This study was conducted in two main phases: 1) development of an instrument for DVRS and 2) pilot implementation of the DVRS in two pilot facilities. Phase one includes developing the minimum datasheet by using inputs from expert panels, content validation of the instrument, and test-retest reliability. In phase two, the datasheet designed in the previous step was used in a pilot implementation of the DVRS for 12 months to find practical challenges. The process of the development and pilot implementation of the DVRS is presented in Fig 1.

### Phase 1: Development of a minimum datasheet

#### Expert panel

In the first step, a preliminary datasheet was developed by using documentary review and expert panels. Data items in similar registry programs [21, 28, 29] and injury surveillance guidelines of WHO [30] were congregated in a preliminary datasheet and then were modified by four panels of experts in social harm, community/public health, epidemiology, emergency medicine, and forensic medicine, who had experience with work on violence. The preliminary datasheet was a closed questionnaire containing different types of questions including multiple and single choice questions and questions with text answers for national codes, residency, and date and time. Participants in all panels were the same and included two emergency medicine specialists from hospitals, one forensic medicine specialist and two general practitioners from the Forensic Medicine Organization, an epidemiologist, two community medicine specialists, and a Ph.D. in health services management from the University. Participants’ minimum work experience was six years, and maximum work experience was 28 years. In the expert panels, no items were removed from the preliminary instrument; only extra items were added, as well as some changes were made in the choice options.

#### Content validation

In this step, validity of the instrument was evaluated by calculating the Content Validity Index (CVI) and Content Validity Ratio (CVR). Using the Delphi technique in two rounds, professionals with vast experience in violence, injury, or social harm evaluated the instrument. They provided their professional opinion on the contents of the instrument, without face-to-face contact, in an iterative manner until a consensus was obtained. In the next round, a summary of the first-round responses was shared with all participants for further explorations. Participants in both rounds of the Delphi were the same individuals.

A total of 22 professionals participated in the Delphi technique. Six were working in the forensic medical clinics, four in the emergency departments, two in the psychiatry clinics, three in the Welfare Organization, and seven in the University. To calculate CVR and CVI, responses from all raters were pooled, separately for CVI and CVR. After receiving the participants’ feedback, the content validity of the items of the instrument was calculated using the Item Content Validity Index (I-CVI) and CVR. The item ratings of I-CVI were on a four-point ordinal scale for three criteria including relevance (1 = not relevant, 2 = somewhat relevant, 3 = quite relevant, and 4 = highly relevant), clarity/understandability (1 = not clear, 2 = somewhat clear, 3 = quite clear, and 4 = highly clear), and simplicity (1 = not simple, 2 = somewhat simple, 3 = quite simple, and 4 = highly simple). The formula used for I-CVI was:

$$I‐CVI=\frac{\mathrm{Number}\;\mathrm{of}\;\mathrm{raters}\;\mathrm{giving}\;\mathrm{rate}\;3\;\mathrm{or}\;4\;\mathrm{to}\;\mathrm{an}\;\mathrm{item}}{\mathrm{Total}\;\mathrm{number}\;\mathrm{of}\;\mathrm{raters}}$$

The item ratings of CVR were on a four-point ordinal scale for the criterion necessity (1 = essential, 2 = useful, 3 = not essential, and 4 = not useful). The CVR formula was:

$$CVR=\frac{n‐(\frac{N}{2})}{\frac{N}{2}}$$

Wherein n is the number of the panelists giving the rate 3 or 4 to an item and N is the total number of panelists.

In each round of the Delphi, the items with CVR≥0.42 [31] and with I-CVI≥0.78 [32] were kept, and 0.3≤CVI≤0.78 moved to the next round. In addition to calculating the CVI score for each item (I-CVI) in each round, the CVI was calculated for the overall scale (S-CVI) [32]. To calculate the S-CVI, the average of the I-CVI was calculated.

#### Test-retest reliability

After testing the validity of the selected items in the previous step, we examined the reliability of the items that seem to result in inconsistent responses. For this purpose, we used the reliability coefficient to quantify the degree of the consistency of the responses in terms of the self-reported items in the instrument. We asked the self-reported items, which we assumed may had been inconsistent, from a sample of 30 victims and asked the same items from them 14 days later. If there was no significant change in response for an item, a reliable item given at these two different times should yield similar results, meaning that the responses were consistent. The Pearson correlation coefficient, r, was used and the formula is presented below:

$$r=\frac{N\sum xy‐(\sum x)(\sum y)}{\sqrt{\left[N\Sigma x^{2}‐{\left(\sum x\right)}^{2}][N\Sigma y^{2}‐{\left(\sum y\right)}^{2}\right]}}$$

Where r is the Pearson correlation coefficient, N is the total number of pairs of test and retest scores; x and y are the test and retest scores respectively. We used Vincent’s benchmarks to interpret Pearson correlation coefficient results, wherein a value of 0.7 and over was considered strong correlation, between 0.5 and 0.7 a moderate correlation, 0.5 and below was considered poor correlation [33].

For test-retest reliability examination, a sample of 30 victims of DV (participants of both test and retest) were approached in an FMC. In each clinical examination, an attended physician provided the patients with information on the DVRS’s objectives and features and requested them to participate in the pilot study. After filling out the informed consent form, if an individual agreed to participate, their information was inserted in the datasheet by a last-year medical student. At a 14 days interval, another investigator contacted each registered individual regarding the self-reported items for the second time. Although 40 individuals were approached in the FMC, ten individuals were not available in the retest step and were excluded. Correlation between test-retest variables, including age/sex (perpetrator), education (victim/perpetrator), occupation (victim/perpetrator), alcohol/drug use (victim/perpetrator), and history of violence, were calculated using SPSS-21.

### Phase 2: Pilot implementation of the DVRS in two pilot facilities

#### Characteristics of the DVRS

**Fig 2:**
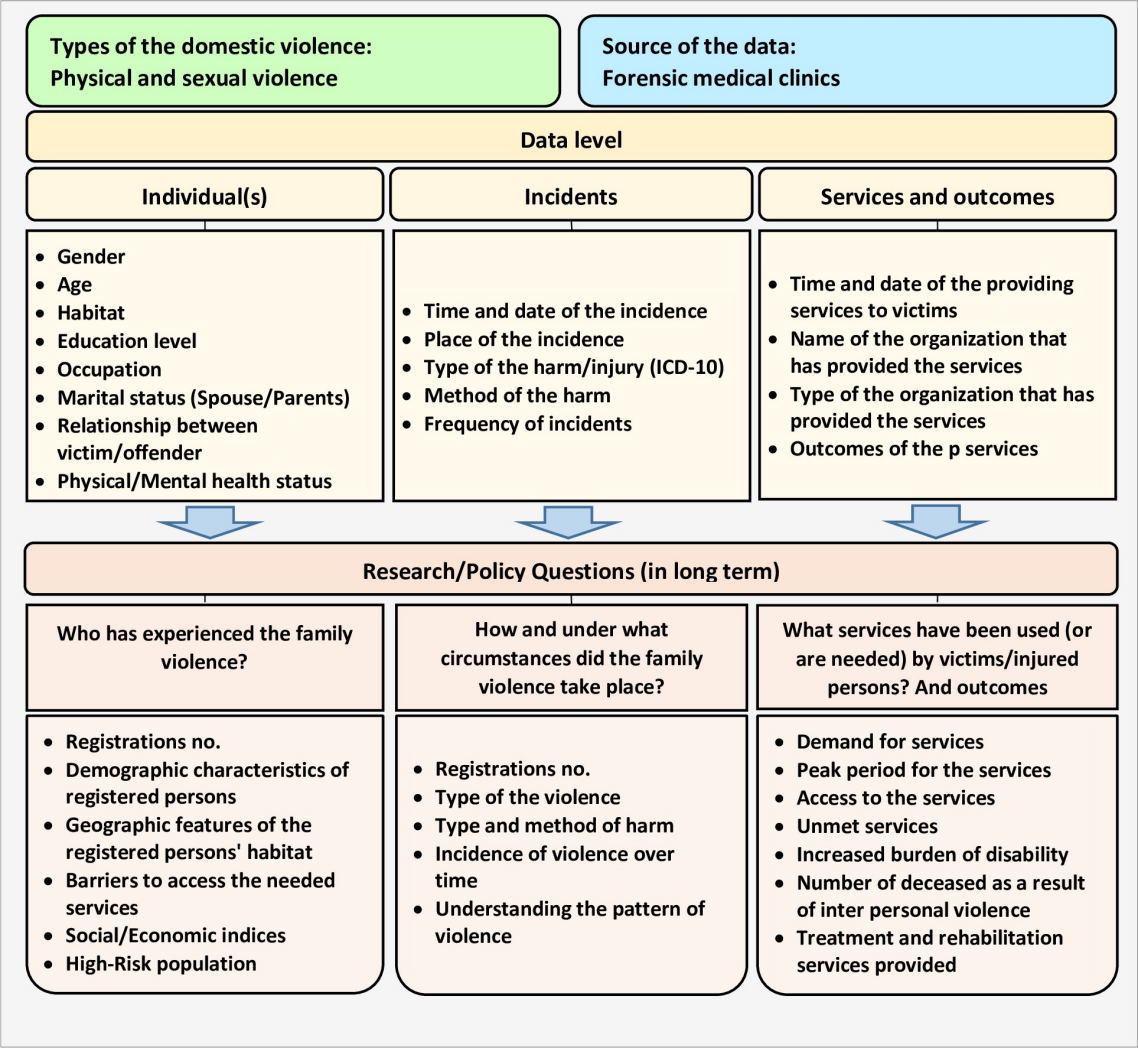
Conceptual framework of the domestic violence registry system in East-Azerbaijan Province of Iran.

In this step, we outlined the elements of the DVRS, including types of domestic violence, data sources, data level, and research/policy questions that can be answered by the registry program, in a conceptual framework. The conceptual framework of the DVRS and characteristics of the registry system was drawn from the opinions of professionals in the prior report [27] and expert panels and are provided in Fig 2. Although when planning for the development of the registry system, the investigators were thinking of a broad and comprehensive interpersonal registry system, after conducting a rigorous qualitative study with the participation of the stakeholders at the province level, the investigators decided to narrow the registry system by targeting family members as the target population and physical and sexual violence as the types of violence.

#### Main outcome

The main outcome of interest in the registry system was among those included in the International Classification of Diseases (ICD 10th revision; S00-T88 Injury, poisoning and certain other consequences of external causes, whose intentionality was confirmed by forensic medical clinics).

#### Data collection

Using the minimum datasheet developed and validated in the previous phase (S1 File), two physicians in two FMCs in two different districts in the EastAzerbaijan province of Iran spent 12 months registering the individuals exposed to physical or sexual violence by a family member. Two of the authors instructed the registrars, and a preliminary protocol of the registry system was provided for them before the pilot implementation. Registrars were the clinics’ attended physicians and conducted the clinical examination of the victims/injured individuals.

#### Eligibility criteria

According to the results of a qualitative study conducted regarding the feasibility of an interpersonal violence registry system in Iran prior to the development of the registry system [27], panelists in the expert panels decided to narrow the registry system by targeting only physical and sexual violence among family members. Their rationale to choose this population and these types of violence was that the most sensitive types of violence, including child abuse, elderly abuse, and domestic violence, occur among this population. These are the most severe types of violence, usually accompanied by other types of violence, such as emotional and financial violence, reported by the victims in Iran [27]. Inclusion and exclusion criteria for registrations are provided in Table 1.

**Table 1:** Eligibility criteria for registration of the family violence cases.

**Inclusion criteria for registration**		
Individuals physically injured by one of their close family members or an acquaintance	**OR**	Victims of sexual violence whose perpetrators were their close family members or an acquaintance
	**AND**	
Individuals reported their violence incidence to one of the pilot forensic clinics.		
**Exclusion criteria**		
Violence incidents not identified and classified as domestic violence by the attending physician		
**AND/OR**		
Violence perpetrated by friends, neighbors, colleagues, classmates, or strangers.		

#### Data analysis

We performed a descriptive analysis of the data. Our main objectives were to see how the DVRS works in a real-world setting and to determine the main issues with the implementation of the program. Descriptive analysis was conducted to report frequency and percentage for categorical variables and mean and standard deviation for continuous variables using SPSS-21.

## Results

The results of this study show the step-by-step process of the development, validation, and pilot implementation of the data collection tool in a DVRS in two main phases.

### Phase one: Development and validation of a minimum datasheet

After conducting a documentary review and four expert panels, preliminary instrument consisted of 31 items in two main categories, including 22 items for individuals’ characteristics (victim and perpetrator) and 9 items for incidents’ characteristics. Finally, after validation and test-retest reliability examinations, 24 items were included in the instrument including 15 items for individuals’ characteristics (victim and perpetrator), 8 items for incidents’ characteristics, and one item for past history of violence experience. Victims’ characteristics included date of birth; residential place specified by province and city/village name; sex categorized as male, female, and unknown; marital status categorized as single, married, divorced, widowed, and other; a multiple-choice question for health status categorized as intellectual disability, physical disability, none, and other/unknown; single choice questions for custody of child (in case), custody of the elderly (in case), education, occupation, offender’s relation with the victim; and yes/no questions for alcohol and drug consumptions. Perpetrators’ characteristics contained age as years of old; sex classified as male, female, and unknown; and three single choice questions for marital status, education level, and occupation. Incidents’ characteristics contained the location of incidence classified as home, school, public place, and other/unknown; time and date of incidence; a single-choice question for victim’s disposition; and multiple-choice questions for the mechanism of injury, the context of incidence, objects used in injury, type of injury, and areas of injury. The results of the validation and the reliability examinations are explained below. The past history violence incidence was a yes/no question and in case the answer was yes, a multiple-choice question asked about the context of the previous incidence.

#### Validation results

Using the Delphi technique, the preliminary instrument was evaluated for content validity. In the first round of Delphi, fifteen items had good content validity (I-CVI and CVR) and were kept, and seven items were moved to the next round. Additionally, in the first round of Delphi, experts suggested adding three items as well, including the history of violence, custody of the child (when the victim is a child), and custody of the elderly (when the victim is elderly). All items evaluated in the second round were kept due to good CVR and CVI scores (Table 2). Additionally, S-CVI estimated 0.93, which was considered good. Scores of the items are presented in Table 2.

**Table 2:** CVI and CVR scores in a two-round Delphi.

Items	Round 1		Round 2	
	CVR	I-CVI	CVR	I-CVI
**Victim characteristics**				
Age	1.00	1.00		
Address*	0.20	0.67		
Sex	1.00	1.00		
Marital status**	1.00	0.58	1.00	0.90
Ethnicity*	0.00	0.58		
Education**	1.00	0.60	1.00	1.00
Occupation **	0.80	0.67	0.80	0.88
Income*	-0.90	0.17		
Health status**	0.80	0.45	0.80	0.87
Relationship with perpetrator	1.00	1.00		
Alcohol use	1.00	1.00		
Drug use	1.00	1.00		
Custody of the child***	-	-	0.90	1.00
Custody of the elderly***	-	-	0.90	1.00
**Incident characteristics**				
Date and time	1.00	0.73		
Place	1.00	0.40	1.00	0.92
Activity at the time of incident*	-0.60	0.25		
Mechanism	1.00	1.00		
Context	1.00	1.00		
Objects used	1.00	0.87		
History of incidence***	-	-	0.80	0.93
**Victims status after the incident**				
Referral status	1.00	0.90		
Types of injury(s)	0.80	0.95		
Injury area(s)	1.00	1.00		
**Perpetrator characteristics**				
Age	1.00	1.00		
Address*	0.00	0.67		
Sex	1.00	0.82		
Marital status*	0.20	0.70		
Ethnicity *	-1.00	0.03		
Education **	1.00	0.63	1.00	0.88
Occupation **	1.00	0.70	1.00	0.88
Income*	-1.00	0.07		
Alcohol use	1.00	1.00		
Drug use	1.00	1.00		

* Items deleted due to low CVI and CVR.

** Items moved to the next round Delphi.

*** Items added in the round 2 Delphi.

#### Test-retest reliability examination results

Test-retest correlation coefficients were high for items including victim/perpetrator education, victim/perpetrator job, victim’s alcohol/drug status, history of violence incidence, and perpetrator’s age and sex. Although for items including perpetrator’s alcohol and drug status, the correlations between test and retest were found to be statistically significant, r(30) = +.43, and +.38, p< .01, two-tailed, respectively, the reliability was unacceptable (< 0.5) and therefore we removed those two items from the datasheet (Table 3).

**Table 3:** Results of the test-retest correlation coefficients.

Items	N (%)	Correlation (two-tailed)	P-value	Decision
Education-Victims	30 (100%)	1.00**	.000	Kept
Occupation-Victims	30 (100%)	.98**	.000	Kept
Alcohol-Victims	30 (100%)	1.00**	.000	Kept
Drug-Victims	30 (100%)	1.00**	.000	Kept
History of violence	28 (93%)	.78**	.000	Kept
Age-Perpetrator	11 (37%)	.98**	.000	Kept
Sex-Perpetrator	28 (93%)	1.00**	.000	Kept
Education-Perpetrator	24 (80%)	.95**	.000	Kept
Occupation -Perpetrator	27 (90%)	.72**	.000	Kept
Alcohol-Perpetrator	30 (100%)	.43*	.017	Removed
Drug-Perpetrator	30 (100%)	.38*	.017	Removed

** Correlation is significant at p<0.01 (2-tailed).

* Correlation is significant at p<0.05 (2-tailed).

### Phase two: Pilot implementation of the DVRS in two pilot facilities

Descriptive results of the cases registered in the DVRS showed that 369 cases were registered from September 23, 2019, to July 21, 2020, and all were from urban areas. The majority of the reported cases were female (82%) and were 19–40 years old. The minimum age of the perpetrators was 21. Additionally, the minimum and maximum ages of the victims were seven and 81 years, respectively. The full report of the results is presented in S2 File.

**Fig 3:**
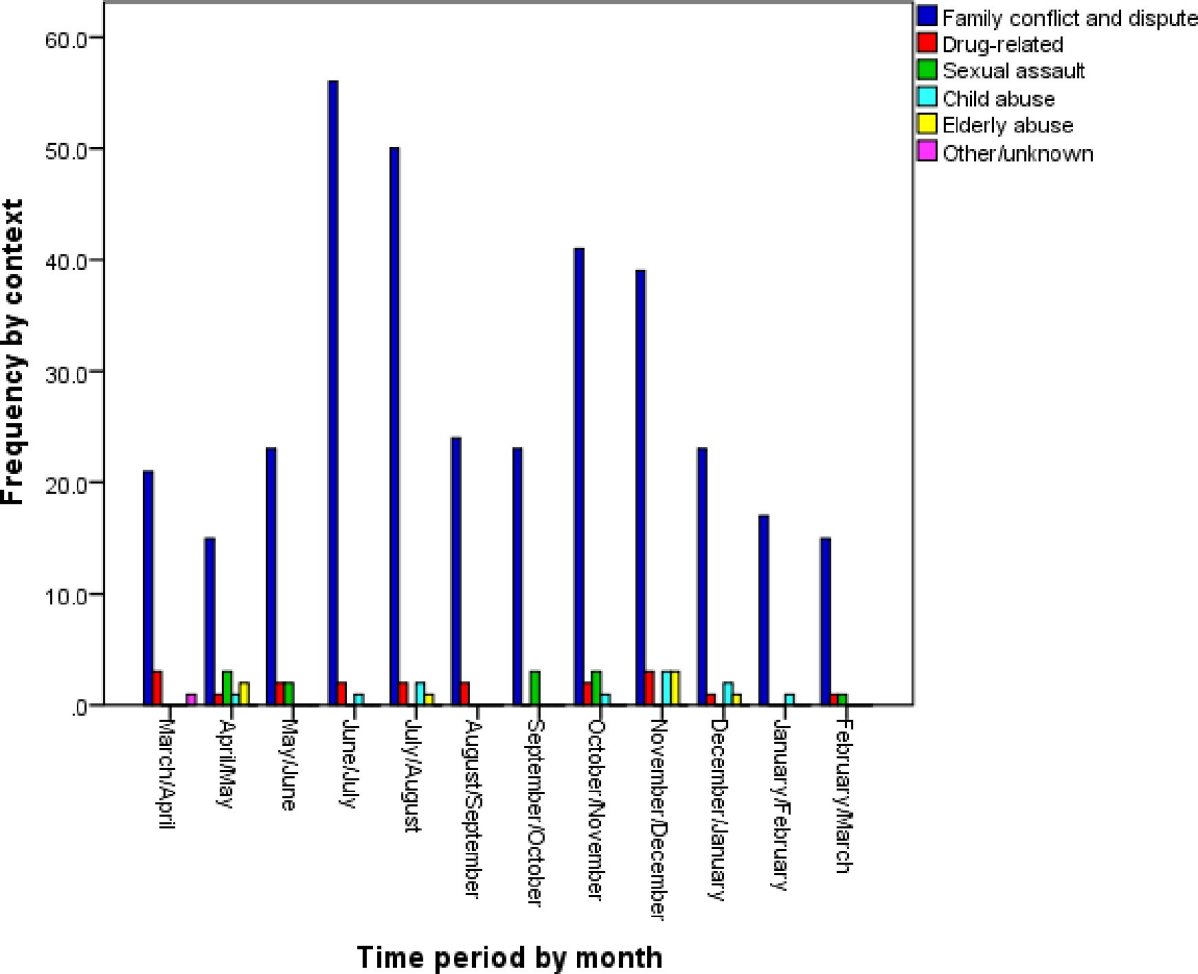
Frequency of domestic violence incidents categorized by the context during the pilot implementation.

**Fig 4:**
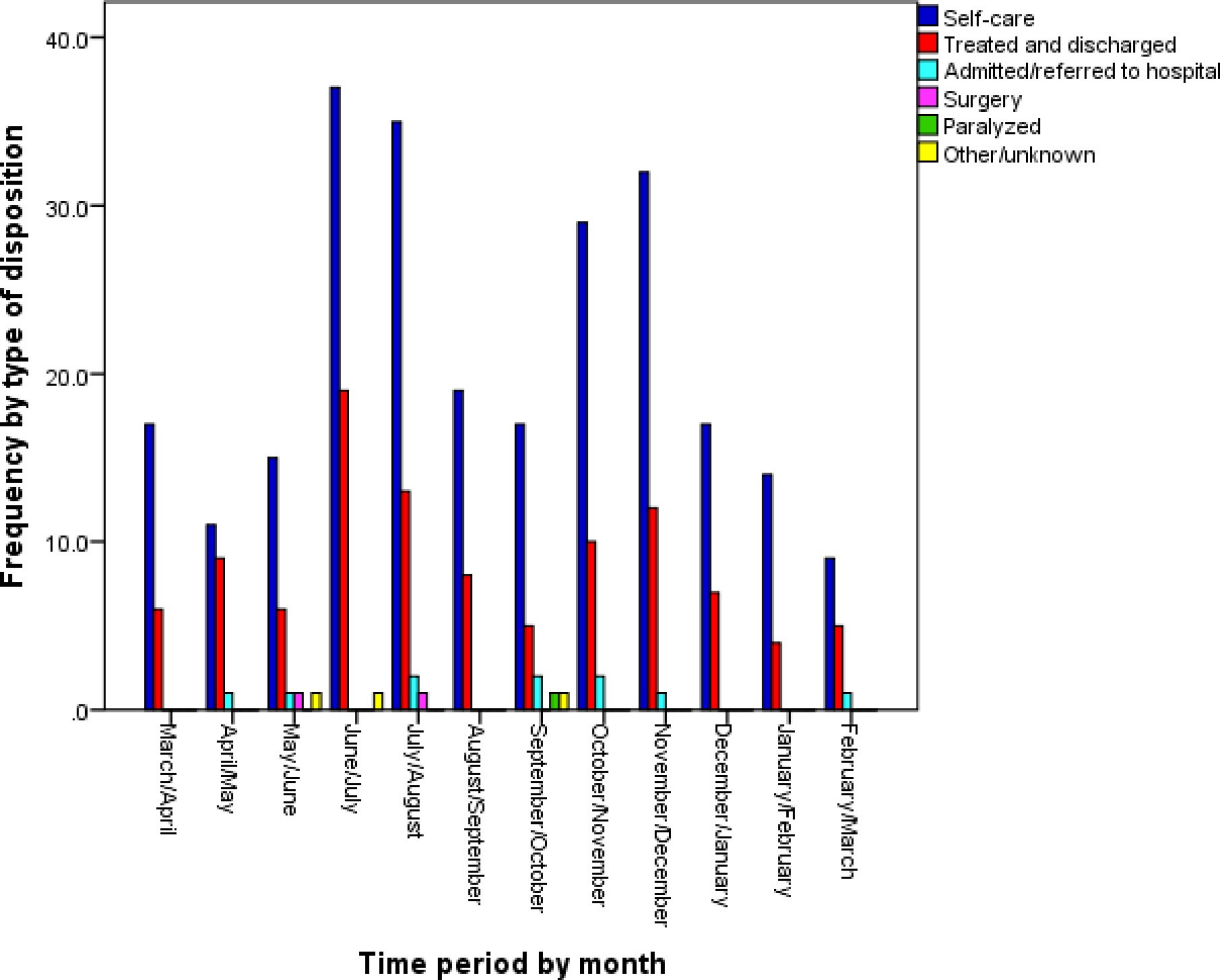
Frequency of domestic violence incidents categorized by victims’ disposition status during the pilot implementation.

**Fig 5:**
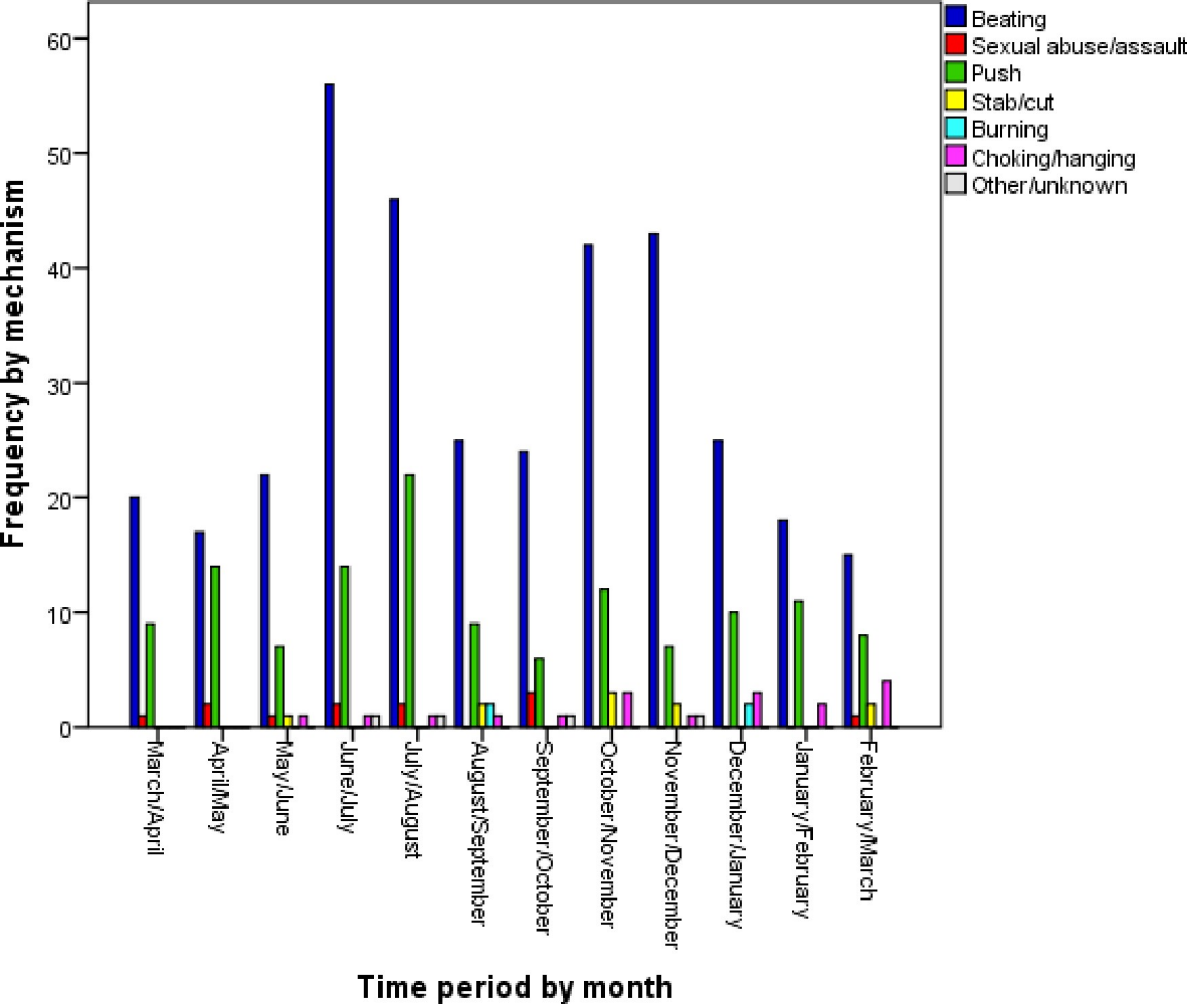
Frequency of domestic violence incidents categorized by the injury mechanism during the pilot implementation.

The main context of the incidents was family conflict and dispute (Fig 3). Regarding the status of the victims after the incidence, results showed that most victims had chosen self-care (68.5%) (Fig 4). Additionally, in most cases, the victim was the perpetrator’s spouse (75%), among which 2.5% were male victims injured by their wife. The mechanism of injury in most of the cases was beating (Fig 5). During the fifth, sixth, and seventh months, no cases were registered in Ahar district. Due to the high volume of work in these periods, registrars could not spend time registering cases. On the other hand, the response rate for the majority of the demographic items was 49%. The DVRS is expected to show a dynamic status of the DV incidences as is presented in Figs 3–5.

## Discussion

A full explanation of a violence registry instrument’s development process is essential to guarantee the successful application of the instrument in a real-world setting. This study outlined the rigorous steps toward developing and validating an instrument to register the physical and sexual DV cases in Iran and pilot-tested it in two FMCs.

Going through a rigorous process of evaluating the validity and reliability of the instrument, such as content validity and test-retest reliability, allowed us to develop a robust instrument to be used in the whole country in a DVRS. A scoping review on child-to-parent violence measures showed that developing a valid and reliable tool to assess the violence is a major prerequisite of conducting research and implementing interventions in this field. Most instruments that assess child-to-parent violence need to be redeveloped and rigorously validated, which calls for developing robust instruments in similar fields [34].

In the first step, we reviewed the literature and evidence in relevant topics and held expert panels to develop the preliminary instrument. Next, we validated the instrument using CVI and CVR through the Delphi technique. A recent study on validating a questionnaire to identify violence in affective relationships has adapted a similar approach [35]. Using the Delphi technique has been shown to be appropriate for similar studies because of its economic feasibility and engagement of qualified professionals in research topics that are new and in their infancy [36].

To assess the reliability of the self-reported items, we conducted a test-retest examination with a 14-day interval. Although, to our knowledge, there is no similar study to develop a registry instrument for interpersonal violence, there are examples of developing instruments for assessing various types of violence at the community level with similar methods to ours. For example, a study conducted a test-retest analysis to assess the reliability of a sexual violence survey [37]. Another similar example is related to Chan and colleagues’ study to develop and validate the family politicization screen [38]. In contrast, in a similar effort in assessing the validity of a sexual experience survey short form, Canan et al. used a five-step mixed-method analysis instead of conventional statistical validity analyses because the short-form survey differs from a simple questionnaire and is an induced model in which individual variables could merge to create a broader category to be examined separately as well [18].

Another feature of the present study that is novel is that the registry program was run for 12 months in two FMCs in separate districts. This is of particular value because it is not easy to initiate such public health intervention in the context of Iran. Additionally, other countries with similar contexts like Afghanistan and Pakistan can take advantage of this effort and use the instrument in their own country after further adaptation. Although the quantitative results of the pilot study could not show the prevalence of DV and its related items in the local area, it will make a significant contribution to highlighting the areas of special needs for intervention and research. This also will help tailor public health efforts of government planning. For example, the results showed that most registrants were women injured by their husbands. Several studies have confirmed that women are disproportionately affected by DV [39–41]. However, the main interpretation of this finding of the registry program could highlight that women are even more able to report their violent experience than children and the elderly in these districts.

Results of this pilot study show that no relevant cases were reported from rural areas. However, evidence shows that disparity in domestic violence in rural areas is a growing public health issue [42]. In considering this, we assume that social and cultural norms that deter the help-seeking behavior of DV victims in developing countries are more potent in smaller rural districts and areas. Evidence corroborates that social and cultural norms of communities in rural areas, particularly in developing countries, result in acceptance of DV, even by women [43, 44]. Further, because women in rural areas are almost always from disadvantaged groups and have less access to support services, they are less likely to seek support and help [42, 45]. Due to the inclusion of only physical and sexual violence that caused an injury, only the women who had severe experiences with violence found this level of violence unacceptable and had access to FMCs reported violence experiences to FMCs.

There remain concerns regarding the rights of children and the elderly. Most physical and sexual violence against children and the elderly are hidden, especially in rural areas. A small amount of evidence shows that because of fear of the perpetrator, fear of not being believed, and not being noticed or asked, children usually do not disclose violent behavior incidences and do not seek violence-response services [46, 47]. Additionally, some studies have highlighted the higher vulnerability of male victims of sexual abuse for reporting the incidence [48, 49]. Although the Welfare Organization in Iran has the responsibility to identify child abuse cases by public-reporting strategies and provides emotional supports (advisory services), it is not clear how effectively this organization acts in rural areas because people in rural areas may not be familiar with such services. On the other hand, women, children, and the elderly may not be able to easily access these services. Therefore, concerns about DV victims in the district and rural areas need to be addressed systematically.

Although the establishment of a DVRS is a promising initiative to address the problem at the community level, there is no guarantee for sustainable development of the program due to the limited available resources. The DVRS faces significant challenges to successful implementation. Because of the high volume of work in particular conditions, such as during the COVID-19 pandemic or in the summer, it is impossible for attending doctors to fill out the datasheets during these times. For example, according to the declarations of the registrars in the FMCs, since the beginning of the COVID-19 epidemics and during the lockdown, for 2–3 months, the frequency of reported cases of DV had increased to the extent that they could not fill the forms for most of the cases. This is a challenge that could affect other regions in the world. Evidence shows that because of the high stress that people experience during the pandemic, and because home is not always a safe place for every family member, women and children are at more risk of domestic violence than pre-pandemic [50]. Therefore, additional trained human resources are needed to do the registries.

Furthermore, attending doctors need financial incentives to conduct registrations as extra work, even during routine and low-volume periods. Using strategies such as pay-for-performance or fee-for-service as financial incentives in the registry program could solve the problem. Nevertheless, this needs financial support. Finally, to avoid missing the complete records of cases in special situations like crises or epidemics, in which the volume of work is higher than usual, and it is challenging for staff and practitioners to fill out time-consuming forms, we indicated necessary items by asterisks (*) (see S1 File).

### Implications of the study

This study can contribute to establishing a DVRS in the whole country and other medical facilities, like hospitals and primary healthcare centers. Such registry systems would have the capability to show trends in DV by age, sex, the context of the violence, and incidence characteristics at every point in time. This is particularly valuable in planning and prioritizing research areas for DV prevention. The instrument developed in this study and the structure of DVRS can be adapted in other countries with similar contexts.

### Limitations

This study has several limitations. First, the registry program is designed to register only cases reported as violent incidences. Hence, unreported cases are missed from the registry program. Considering the social and cultural context of Iran, it is not possible to include all victims in the program, and only cases reported by the victims can be identified [27]. Evidence suggested that registry data does not represent all cases [51]. Thus, we plan to develop a DV screening program for school settings and primary health centers which can result in further coverage of the program. Second, DV can encompass a wide range from emotional to physical types. Nevertheless, we have only included physical and sexual violence in the program. This is due to the opinions of the professionals in the feasibility study [27]. As mentioned above, considering the limited resources and infrastructures, as well as social norms, it is not feasible to register all types of DV. Third, despite the high reported DV cases at the beginning of the COVID-19 pandemic, we missed many cases due to the high volume of work in the FMCs. Registrars declared that they could not register all DV cases from January 21 to April 17, 2020 (third period of the registry) due to the high volume of work in the FMCs. Fourth, the pilot test was conducted in FMCs, and we excluded hospitals. In this step, we wanted to test the DVRS with the most accurate data to find the design and implementation gaps. Clients of the FMCs explicitly report violent experiences, and the authorities confirm the intentionality of the harm. Therefore, FMCs can provide the most valid and reliable data for the registry program in this step. In the next step, we are going to run the program in both FMCs and hospitals.

## Conclusion

In this study, we developed a valid and reliable instrument to register the physical and sexual DV cases in a DVRS that various professionals can easily use in various organizations. A pilot study in two FMCs showed that no physical and sexual violence was reported from rural areas, which calls upon researchers to explore ways in which services and supports for detecting and treating victims can be made accessible to these vulnerable groups. Additionally, a 12-month pilot implementation of the registry system showed that although establishing a DVRS could be a promising and effective initiative in identifying the areas in need of urgent intervention, there is no guarantee for sustainable implementation of the program due to lack of adequate human resources and financial incentives for registrars. It is therefore essential to find a sponsor for the program before implementing it on a larger scale.

## Supporting information

S1 File Domestic violence registry datasheet. (DOCX)

S2 File Descriptive report of the registered cases (N = 369). (DOCX)

S1 Data (SAV)
