# Supplementary material for: Development, validation, and pilot implementation of the minimum datasheet for a domestic violence registry system: The case of a developing country
Source: PLoS One. 2021 Dec 31;16(12):e0261460. doi: 10.1371/journal.pone.0261460 (PMC8719697; doi:10.1371/journal.pone.0261460)
Supplement: S1 File — (DOCX) [file pone.0261460.s001.docx]

**S1 File. Domestic Violence Registry datasheet**

|  |  | / |  |  | / |  |  | date |  |  | : |  |  | Time | Registration No. : |
| --- | --- | --- | --- | --- | --- | --- | --- | --- | --- | --- | --- | --- | --- | --- | --- |

**Part 1) Victim information**

National ID: …………………………………….. Tell: ……………………………………………..

| Day | | / | Month | | / | Year | | | | Date of birth |
| --- | --- | --- | --- | --- | --- | --- | --- | --- | --- | --- |
|  |  | / |  |  | / |  |  |  |  |  |

| City or village: | Province: | Residency |
| --- | --- | --- |

*Sex: Male 🗆 Female 🗆 Unknown🗆

| *Marital status: (If the child or elderly is injured, the marital status of the head of the family): | | | | |
| --- | --- | --- | --- | --- |
| Other 🗆 | Widowed🗆 | Divorced 🗆 | Married 🗆 | Single 🗆 |

| *Custody of child | | | | |
| --- | --- | --- | --- | --- |
| Other/unknown 🗆 ……………………… | Other families 🗆 | Mother 🗆 | Father 🗆 | Parents 🗆 |

| *Custody of the elderly | | | |
| --- | --- | --- | --- |
| Nursing home 🗆 | offspring 🗆 | Alone 🗆 | Living with a spouse 🗆 |
| Other/unknown 🗆 ……………………… | | Other families 🗆 | Offspring🗆 |

| Education: | | | |
| --- | --- | --- | --- |
| 9-7Class 🗆 | 4-6Class 🗆 | 1-3Class 🗆 | Illiterate 🗆 |
| Bachelor 🗆 | Upper Diploma 🗆 | Diploma 🗆 | 10-12Class 🗆 |
|  | Unknown 🗆 | Doctorate 🗆 | Master 🗆 |

| Occupation: | | | |
| --- | --- | --- | --- |
| Self-Employed 🗆 | Retired 🗆 | Private Employee 🗆 | Government Employee 🗆 |
| Homemaker 🗆 | University Student | Student 🗆 | Worker 🗆 |
| Other 🗆 ………………………………….. | | Unknown 🗆 | Unemployed 🗆 |

| health status: | |
| --- | --- |
| Intellectual disability 🗆 | Physical disability 🗆 |
| Other/ Unknown 🗆 ……………………………. | None 🗆 |

| *Offender’s relation with the victim | | | | |
| --- | --- | --- | --- | --- |
| Offspring 🗆 | Step-Parent 🗆 | Parent 🗆 | Ex-spouse 🗆 | Spouse 🗆 |
| Unknown 🗆 | Other Families 🗆 | Step-offspring 🗆 | Sibling 🗆 | Step-offspring 🗆 |

| Alcohol consumption: Have you consumed alcohol in the last 6 hours (before incidence)? | |
| --- | --- |
| Yes 🗆 | No 🗆 |

| Drug consumption: Have you consumed drug in the last 6 hours (before incidence)? | |
| --- | --- |
| Yes 🗆 | No 🗆 |

**Part 2) incidence**

| Location: Where were you at the time of the incidence? | | | |
| --- | --- | --- | --- |
| Other/unknown 🗆 ……………………… | Public Place 🗆 | School 🗆 | Home 🗆 |

|  |  | / |  |  | / |  |  | date |  |  | : |  |  | *Time |
| --- | --- | --- | --- | --- | --- | --- | --- | --- | --- | --- | --- | --- | --- | --- |

| Mechanism: How did you get injured? | | | | |
| --- | --- | --- | --- | --- |
| Stab/cut 🗆 | Push 🗆 | Biting 🗆 | Sexual abuse 🗆 | Beat 🗆 |
| Other/unknown 🗆 ………………………………. | | Shooting 🗆 | Choking/hanging 🗆 | Burn 🗆 |

| *Context | | |
| --- | --- | --- |
| Child abuse 🗆 | Drug-related 🗆 | Quarrel 🗆 |
| Other/unknown 🗆 : ……………………………. | Elderly abuse 🗆 | Sexual Assault 🗆 |

| Object used | | | |
| --- | --- | --- | --- |
| Fire/hot object 🗆 | Knife/cutting tool 🗆 | Club/stick 🗆 | Blunt Force 🗆 |
| Other/unknown 🗆 : …………… | Gun 🗆 | Acid 🗆 | Fire/hot liquid 🗆 |

| *Disposition | | | |
| --- | --- | --- | --- |
| Undergone Surgery 🗆 | Admitted/referred to hospital 🗆 | Treated and discharged 🗆 | Self-care 🗆 |
| Other/unknown 🗆 : ……….. | Hospitalized in ICU 🗆 | Died 🗆 | To be paralyzed 🗆 |

| *Type of injury | | | | |
| --- | --- | --- | --- | --- |
| Burn 🗆 | Bruise 🗆 | Cut, bite, open wound 🗆 | Sprain/Strain 🗆 | Fracture 🗆 |
| Other/unknown 🗆 : ………………. | | Organs system injury 🗆 | Spinal cord injury 🗆 | Brain tissue damage🗆 |

| Injury area | | | | |
| --- | --- | --- | --- | --- |
| Legs, knees, femurs 🗆 | Hip 🗆 | Hands, arms, elbows 🗆 | Shoulder 🗆 | Head and neck 🗆 |
| Nose 🗆 | Ear 🗆 | Eye 🗆 | Face 🗆 | Sex organ 🗆 |
| Other/unknow 🗆 : ………… | Stoma | Breast 🗆 | Waist 🗆 | Mouth/ teeth 🗆 |

**Part 3) History of violence**

| Is this your first experience? No 🗆 Yes 🗆 | | |
| --- | --- | --- |
| If yes; what was the context of the previous incidence: | | |
| Child abuse 🗆 | Drug-related 🗆 | Quarrel 🗆 |
| Other/unknown🗆 : ……………………………. | Elderly abuse 🗆 | Sexual Assault 🗆 |

**Part 4) Perpetrator information**

Age: ………………….. *Sex: Male 🗆 Female 🗆 Unknown 🗆

| Marital status: (If the child or elderly person is injured, the marital status of the head of the family): | | | | |
| --- | --- | --- | --- | --- |
| Other 🗆 | Widow/Widower 🗆 | Divorced 🗆 | Married 🗆 | Single 🗆 |

| Education: | | | |
| --- | --- | --- | --- |
| 9-7Class 🗆 | 4-6Class 🗆 | 1-3Class 🗆 | Illiterate 🗆 |
| Bachelor 🗆 | Upper Diploma 🗆 | Diploma 🗆 | 10-12Class 🗆 |
|  | Unknown 🗆 | Doctorate 🗆 | Master 🗆 |

| Occupation: | | | |
| --- | --- | --- | --- |
| Self-Employed 🗆 | Retired 🗆 | Private Employee 🗆 | Government Employee 🗆 |
| Homemaker 🗆 | University Student 🗆 | Student 🗆 | Worker 🗆 |
| Other 🗆 …………………………………… | | Unknown 🗆 | Unemployed 🗆 |
