# Supplementary material for: Development, validation, and pilot implementation of the minimum datasheet for a domestic violence registry system: The case of a developing country
Source: PLoS One. 2021 Dec 31;16(12):e0261460. doi: 10.1371/journal.pone.0261460 (PMC8719697; doi:10.1371/journal.pone.0261460)
Supplement: S2 File — (DOCX) [file pone.0261460.s002.docx]

**S2 File. Descriptive report of the registered cases (N=369)**

| Characteristics | N | % |
| --- | --- | --- |
| Sex | 368 |  |
| Male | 65 | 18 |
| Female | 303 | 82 |
| Age group | 171 |  |
| 0-18-Years | 12 | 7.0 |
| 19-40-Years | 111 | 64.9 |
| 41-60-Years | 42 | 24.6 |
| 61-above | 6 | 3.5 |
| Marital Status | 364 |  |
| Single | 18 | 4.9 |
| Married | 321 | 88.2 |
| Divorced | 13 | 3.6 |
| Widowed | 10 | 2.7 |
| Other | 2 | .5 |
| Child Custody | 3 |  |
| Parents | 3 | 100 |
| Elderly Custody | 3 |  |
| Alone | 1 | 33 |
| Offspring | 1 | 33 |
| Education of Victim | 178 |  |
| Illiterate | 14 | 7.9 |
| Elementary | 79 | 44.4 |
| Diploma | 41 | 23 |
| Undergraduate | 35 | 19.7 |
| Graduate | 9 | 5 |
| Occupation of Victim | 172 |  |
| Employee | 16 | 9.3 |
| Self-Employed | 23 | 13.4 |
| Manual worker | 5 | 2.9 |
| Student | 4 | 2.3 |
| Homemaker | 106 | 61.6 |
| Retired | 6 | 3.5 |
| Unemployed | 6 | 3.5 |
| Unknown | 6 | 3.5 |
| Health Status of Victim | 178 |  |
| Physical disability | 4 | 2.2 |
| No disability | 152 | 85.4 |
| Unknown | 22 | 12.4 |
| Relationship | 367 |  |
| Spouse | 274 | 74.7 |
| Ex-spouse | 12 | 3.3 |
| Parent | 10 | 2.7 |
| Step-parent | 2 | .5 |
| Offspring | 5 | 1.4 |
| Step-offspring | 2 | .5 |
| Sibling | 14 | 3.8 |
| Step-siblings | 4 | 1.1 |
| Families | 43 | 11.7 |
| Unknown | 1 | .3 |
| Place of Incidence | 177 |  |
| Home | 141 | 79.7 |
| School | 1 | .6 |
| Public Place | 29 | 16.4 |
| Other/unknown | 6 | 3.4 |
| Mechanism | 368 |  |
| Beating | 354 | 96.2 |
| Sexual abuse/assault | 12 | 3.3 |
| Push | 129 | 35.1 |
| Stab/cut | 10 | 2.7 |
| Burning | 4 | 1.1 |
| Choking/hanging | 18 | 4.9 |
| Other/unknown | 4 | 1.1 |
| Context | 368 |  |
| Family conflict and dispute | 348 | 94.6 |
| Drug-related | 19 | .2 |
| Sexual assault | 12 | 3.3 |
| Child abuse | 11 | 3 |
| Elderly abuse | 7 | 1.9 |
| Other/unknown | 2 | .6 |
| Object Used | 368 |  |
| Blunt Force | 325 | 88.3 |
| Club/stick | 176 | 47.8 |
| Knife/cutting tool | 16 | 4.3 |
| Fire/hot material | 6 | 1.7 |
| Other/unknown | 13 | 3.5 |
| Disposition | 368 |  |
| Self-care | 252 | 68.5 |
| Treated and discharged | 104 | 28.3 |
| Admitted/referred to hospital | 11 | 3 |
| Surgery | 2 | .5 |
| Paralyzed | 1 | .0 |
| Other/unknown | 3 | .8 |
| Type of injury | 368 |  |
| Fracture | 10 | 2.7 |
| Sprain/Strain | 11 | 3 |
| Cut, bite, open wound | 45 | 12.2 |
| Bruise | 295 | 80.2 |
| Cucussion | 2 | .5 |
| Burn | 7 | 1.9 |
| Organs system injury | 22 | 6 |
| Other/unknown | 115 | 31.3 |
| Injury area | 368 |  |
| Head and neck | 155 | 42.1 |
| Shoulder | 51 | 13.9 |
| Hands, arms, elbows | 183 | 49.7 |
| Hip | 7 | 1.9 |
| Legs, knees, femurs | 100 | 27.2 |
| Sex organ | 5 | 1.4 |
| Face | 102 | 27.7 |
| Eye | 66 | 17.9 |
| Ear | 37 | 10.1 |
| Nose | 19 | 5.2 |
| Mouth/ teeth | 33 | 9 |
| Waist | 35 | 9.5 |
| Breast | 33 | 9 |
| Stoma | 23 | 6.3 |
| Other/unknown | 8 | 2.2 |
| History | 121 |  |
| experience of violence | 104 | 85.2 |
| Family conflict and dispute | 99 | 95.2 |
| Drug-related | 7 | 6.8 |
| Child abuse | 3 | 2.9 |
| Elderly abuse | 2 | 1.9 |
| Other/unknown | 1 | 1 |
| Perpetrator's Sex | 367 |  |
| Male | 354 | 96.5 |
| Female | 13 | 3.5 |
| Perpetrator's Age | 118 |  |
| 19-40-Years | 81 | 68.6 |
| 41-60-Years | 34 | 28.8 |
| 61-above | 3 | 2.5 |
| Perpetrator's Education | 124 |  |
| Illiterate | 5 | 4.0 |
| Elementary | 60 | 48.4 |
| Diploma | 25 | 20.2 |
| Undergraduate | 31 | 25 |
| Graduate | 3 | 2.4 |
| Perpetrator's Occupation | 111 |  |
| Employee | 18 | 10 |
| Self-Employed | 49 | 27.4 |
| Manual worker | 24 | 13.4 |
| Student | 1 | .6 |
| Homemaker | 7 | 3.9 |
| Retired | 4 | 2.2 |
| Unemployed | 8 | 4.5 |
| Other/Unknown | 3 | 1.7 |
